# On the Treatment of Asthma

**Published:** 1872-07

**Authors:** George Gaskoin

**Affiliations:** Surgeon to the British Hospital for Diseases of the Skin


*On the Treatment of Asthma.* By George Gaskoin, Esq., Surgeon to the British Hospital for Diseases of the Skin.

In the summer of 1870, I was summoned to a lady suffering from an acute attack of asthma. For several nights she had been restricted to the sitting posture, bent over a table, with the forehead resting on her hands. The distress was very great indeed. She was subject to frequent attacks of the kind, complicated to a very moderate extent with catarrh and bronchitic exudation. Her physician, a gentleman who holds high professional rank, was out of town. Nothing had been omitted in the treatment, which of late was simply palliative. She was recognized as constitutionally asthmatic, and little hope was entertained of permanent amendment. The asthma first occurred on the subsidence of nervous symptoms a few years previous. It had not, as far as I am aware, any marked organic basis. There was observable on the legs an eczematous eruption. Under these circumstances, I directed that the chloroform liniment of the *British Pharmacopœia* should be rubbed briskly into the chest for an hour's space, if possible; and this was done daily by a very efficient attendant, who had sufficient intelligence to comprehend and carry out the treatment. Very early much relief was experienced. On the return of her physician to town at the end of three days, she was already so very much changed for the better that he directed the treatment to be continued. From that time it consisted in the daily repetition of the rubbing process for a month or nearly so, without aid from medicine, and with little restriction as to diet. Beyond the information I received that she was daily improving, I had really little or nothing to do with her professionally, after one or two visits. Under the hands of her attendant, she speedily got rid of the asthma. The patient went out of town in the autumn, and enjoyed perfect health and spirits. She took much walking exercise, with exposure in the cold of the ensuing winter; and, what is very singular, two years have since elapsed with no return of the asthma.

I shall now make a few observations on this method of treatment. For some years, in Paris, asthmatics have been in the habit of resorting to a rubber in the Boulevard Saint Michel, a certain Widow Pau, who pursues there very much the method which I have laid down, only that her nostrum is a secret. She is resorted to by a few wealthy people from this country, and has honorable mention in some of our West End clubs. At the end of the treatment, her patients are presented with a little book or *brochure* containing her successes, which may be said to be fairly written for a book of its class. The cure is apt to disappoint for a few days; but generally great benefit will be found in a fortnight, or even in less time. There is a hint that it is best suited to cases with catarrhal and bronchitic complication. The instance which I have here brought forward seems exactly to correspond with those which are boasted of and detailed historically by Madame Pau.

Before giving directions as to how this treatment should be carried out, I will speak as to the *rationale*. Counter-irritation, especially by blister, issue, and moxa, are of such well-established repute in the treatment of asthma, that I need not dwell on them; but, besides this, a jolting vehicle, anything that leads to displacement of the air stagnant in the vesicles, is proved to give relief in many instances. I should advise, then, that the frictions should be made with such roughness as the case admits. Slight blows with the palm of the hand or the end of a towel on the ribs, are quite allowable; and the friction should be extended to the front of the neck at the lower part, where the vagi enter the chest. I do not think that the composition of the liniment need trouble us, provided it be warm and work easily. Anything like Roche's embrocation would answer very well.

I am not without some experience of asthma, and I am persuaded that the present method will be found a valuable addition to our therapeutic means. If proved not to be novel, it must be conceded that it has fallen into utter neglect.—*British Medical Journal, March,* 1872.

